# Single-cell RNA sequencing unravels T cell exhaustion underlying the chronicity of chromoblastomycosis

**DOI:** 10.3389/fimmu.2026.1784450

**Published:** 2026-04-17

**Authors:** Kexin Lei, Jie Tian, Lu Zhang, Zhuoqing Gong, Wenjie Liu, Zhe Wan, Yang Wang, Ruoyu Li, Bilin Dong, Xiaowen Wang

**Affiliations:** 1 Department of Dermatology and Venerology, Peking University First Hospital, Beijing, China; 2 Research Center for Medical Mycology, Peking University, Beijing, China; 3 National Clinical Research Center for Skin and Sexually Transmitted Diseases, Beijing, China; 4 Beijing Key Laboratory of Innovative Clinical Research and Translation in Skin Diseases, Beijing, China; 5 Department of Dermatology, Hubei Province & Key Laboratory of Skin Infection and Immunity, Center for Infectious Skin Diseases, Wuhan No.1 Hospital, Wuhan, China

**Keywords:** chromoblastomycosis, chronic fungal infection, LAG-3, PD-1, single-cell RNA sequencing, T cell exhaustion, TIM-3

## Abstract

**Introduction:**

Chromoblastomycosis (CBM) is a chronic, neglected tropical fungal infection. Its immunopathogenesis, particularly the mechanism underlying its chronicity, remains poorly understood.

**Methods:**

We performed single-cell RNA sequencing (scRNA-seq) on lesional skin from a CBM patient, followed by comprehensive bioinformatics analyses. We then used multiplex immunofluorescence (mIF) to validate CD4^+^ T cell exhaustion in CBM patient lesions and the mouse model of *Fonsecaea pedrosoi* infection.

**Results:**

We identified a significantly expanded population of exhausted CD4^+^ T cells within the patient’s lesions, which exhibited high co-expression of inhibitory receptors (PD-1, TIM-3, LAG-3) and functional impairment. Trajectory inference suggested a differentiation path from naive towards exhaustion within the chronic inflammatory environment. Cell-cell communication analysis implicated monocytes/macrophages (MoMacs) as key drivers of this process via persistent antigen presentation and ligand-receptor interactions such as CTLA4-CD80/86 and LGALS9-CD44. The accumulation of exhausted CD4^+^ T cells was confirmed in human CBM lesions by multiplex immunofluorescence (mIF), and the progressive development of exhaustion was recapitulated in the mouse model of *Fonsecaea pedrosoi* infection.

**Discussion:**

Our findings establish CD4^+^ T cell exhaustion as an important mechanism underlying the chronicity of chromoblastomycosis, revealing a new immunopathological perspective for this neglected disease.

## 1 Introduction

Chromoblastomycosis (CBM) is a chronic, progressive subcutaneous mycosis caused by various dematiaceous fungi including *Fonsecaea pedrosoi (F. pedrosoi)*, *Cladophialophora carrionii (C. carrionii)*, and *Fonsecaea monophora (F. monophora)*. The World Health Organization classifies CBM as a neglected tropical disease (1, 2). Early surgical excision and antifungal therapy can achieve clinical cure in some patients. However, ineffective treatment often leads to the progression of skin lesions into extensive scarring, ulceration, and joint deformities, causing permanent functional disability and creating a socioeconomic burden (3–5).

The pathogenic mechanisms of CBM are complex, and the chronicity is closely linked to both the pathogen and host immune responses. Factors that confer increased pathogenicity in CBM include thermotolerance, the presence of muriform cells, cell adhesion, hydrophobicity, and melanin production (6). The muriform cell is considered hallmark of CBM disease chronicity, which is believed to be associated with a robust granulomatous response and immune evasion. Moreover, the cytokine profile in patients correlates with disease severity, characterized by Th1/Th2 immune dysregulation, elevated IL-10 secretion, reduced IFN-γ levels, and impaired T-cell proliferation in severe cases (7).

T cell exhaustion is a differentiated state observed during chronic infections where persistent antigen exposure and chronic T cell receptor (TCR) stimulation lead to diminished effector function. Exhausted T cells express high levels of inhibitory receptors, such as PD1, TIM3, LAG3, CTLA4, and TIGIT, display reduced proliferative capacity, and exhibit altered transcriptional and epigenetic profiles, including involvement of the transcription factor TOX. This state results in a stalemate where T cells loss the function and fail to clear the infection effectively (8).

Dysfunctional CD4^+^ T cell responses have been observed in several chronic viral infections, including Human Immunodeficiency Virus (HIV) (9, 10) and Hepatitis C Virus (HCV) (11, 12). Recent studies on the tumor microenvironment have revealed that tumour-specific cytotoxic CD4^+^ and CD8^+^ T cells also exhibit a similar dysfunctional state, characterized by high expression of immune checkpoint molecules, reduced proliferative potential, and decreased cytokine production (13, 14). The development of T cell exhaustion is regulated by complex interactions among multiple transcription factors, epigenetic modifications, and environmental factors. In chronic viral infection and tumors highlight critical roles for transcription factors, such as NFAT, TOX, and NR4A1 in establishing and maintaining the Tex cell phenotype (15–21), while microenvironmental signals, including cytokines and metabolites, also modulate this process (22).

However, the mechanism underlying the chronicity of chromoblastomycosis remains unclear. In this study, we identified CD4^+^ T cell exhaustion in patients with chromoblastomycosis and validated these findings using a mouse infection model. Our results suggest that interactions between MoMacs and T cells may play a role in the development of this exhausted state.

## 2 Materials and methods

### 2.1 Ethical statement

This study was approved by the Clinical Research Ethics Committee of Peking University First Hospital, Beijing, China. We adhered to the guidelines stated in the Belmont Report and those set forth by the Council for International organizations of Medical Sciences in experimental use of animals. All procedures performed in the present study involving the animals (C57BL/6n mice) complied with the guidelines for humane use of laboratory animals from the National Institute of Health and were in accordance with the ethical standards of the institution at which the study was conducted (the Institutional Animal Care and Use Committee of Peking University First Hospital, project permit number: J2024003). All patients provided written informed consent before participation.

### 2.2 Single-cell RNA sequencing and data processing

Specimens were obtained from the Department of Dermatology at our hospital, including skin tissue from one patient with CBM and one control sample of histologically normal skin adjacent to benign nevi. Immediately after resection, each sample was immersed in MACS Tissue Storage Solution (Miltenyi Biotec; cat. no. 130-100-008) on ice and delivered to the laboratory within 45 min for immediate processing.

Single-cell suspensions were prepared from full-thickness skin fragments (~1.0 × 1.0 cm). Tissues were rinsed three times in PBS, followed by an initial enzymatic dissociation using 0.125 mg/mL trypsin at 37 °C for 15 min. The partially dissociated material was then quenched and washed with PBS supplemented with 2% FBS, collected into tubes, and centrifuged at 1000 rpm for 3 min. After removal of the supernatant, the pellet was resuspended in PBS with 2% FBS. Residual tissue that remained undigested was finely chopped and subjected to a second digestion step in a cocktail consisting of 2 mg/mL collagenase IV, 2.5 mM CaCl_2_, 0.1 mg/mL elastase, 2% FBS, and 10 μg/mL DNase I (Sigma; cat. no. DN25-100MG), with continuous shaking for 30 min. Cells were subsequently collected by centrifugation, resuspended in PBS containing 2% FBS, and sequentially passed through 70 μm and 40 μm strainers to remove aggregates and debris.

Cell yield and quality were quantified using the Countstar Rigel S2 (Alit Biotech, Shanghai, China). Viability was determined by trypan blue exclusion on the same platform, and only preparations with >80% viable cells were advanced to scRNA-seq library construction using the 10x Genomics Chromium workflow.

### 2.3 Single-cell capture, library construction, sequencing, and cell ranger processing

Single-cell suspensions were adjusted to a final concentration of 800–1000 viable cells/μL, as measured using the Countstar system, prior to loading onto the Chromium Single Cell Controller (10x Genomics). Libraries were generated using the Single Cell 3′ Library and Gel Bead Kit v3.1 (10x Genomics; cat. no. 1000075) together with the Chromium Single Cell B Chip Kit (10x Genomics; cat. no. 1000074), following the manufacturer’s protocol for droplet-based partitioning and formation of gel bead-in-emulsions (GEMs). Cells were maintained in PBS supplemented with 0.04% bovine serum albumin (BSA). Approximately 10,000 cells were loaded per channel, with an intended recovery of ~10,000 cells.

Within each GEM, cells were lysed and mRNA molecules were barcoded and reverse-transcribed. Reverse transcription was performed on an S1000™ Touch Thermal Cycler (Bio-Rad) under the following conditions: 53 °C for 45 min, 85 °C for 5 min, then held at 4 °C. cDNA was subsequently amplified according to the kit instructions, and cDNA quality was assessed using an Agilent 4200 system (CapitalBio Technology, Beijing, China). Final libraries were sequenced on an Illumina NovaSeq 6000 platform using paired-end 150 bp (PE150) reads, with a minimum depth of 100,000 reads per cell (CapitalBio Technology).

Raw sequencing output files were processed using Cell Ranger (v7.0.1; 10x Genomics). Briefly, base call files were demultiplexed and converted to FASTQ format using cellranger mkfastq, followed by cellranger count for read alignment, filtering, and quantification of cell barcodes and UMIs. Reads were aligned to the GRCh38 (2020-A) human reference, and feature-barcode matrices were generated as input for downstream analyses.

### 2.4 Data preprocessing, quality control, and integration

Single-cell transcriptomic datasets were processed and analyzed in R using Seurat (v5.3.0). Gene–cell UMI count matrices generated by Cell Ranger were imported into R and organized as Seurat objects for downstream preprocessing. To minimize artifacts caused by ambient RNA, each sample was first corrected using SoupX (v1.6.1). Potential doublets were then detected with Scrublet (v0.2.3) and removed. In addition, a small number of cells not flagged by Scrublet but exhibiting concurrent expression of canonical markers from multiple lineages were excluded based on marker-profile inspection.

Cell-level quality filtering was applied to retain high-confidence single-cell profiles. Specifically, we excluded cells with <200 detected genes (low-complexity profiles consistent with empty droplets), cells with >6,000 detected genes (enriched for multiplets), and cells with mitochondrial transcript fractions >15% (indicative of stressed or dying cells). After these filtering steps, the final dataset comprised 21,222 cells and 30,376 genes.

Following QC, data were normalized and scaled in Seurat with regression of mitochondrial content and cell-cycle scores (percent.mt, S.Score, and G2M.Score). Batch effects across samples were corrected using Seurat’s scVIIntegration, and the resulting scVI-derived integrated embedding was used for downstream dimensionality reduction and clustering. Differential expression analyses were performed on the normalized gene expression matrix based on the identified clusters.

### 2.5 Dimensionality reduction, clustering, and cell type annotation

After integration, dimensionality reduction was performed to capture the major sources of biological variation. We applied principal component analysis (PCA) followed by uniform manifold approximation and projection (UMAP) to visualize cellular relationships in a low-dimensional space. Cell neighborhoods were constructed in the reduced space, and clustering was carried out to identify transcriptionally distinct populations.

Cluster-specific marker genes were identified to support cell identity assignment. Cell types were annotated by combining (i) differential marker expression patterns and (ii) established canonical lineage markers from prior literature. The major cell identities were defined using the following marker sets: T cells (*TRAC, CD247, TRBC2, CD4, CD8A, CD8B*), neutrophils (*FCGR3A, CD177, ITGAM, CEACAM8*), B cells (*MS4A1, CD19, CD79A, CD79B*), pericytes (*ACTA2, TAGLN, RGS5, NOTCH3*), melanocytes (*MITF, MLANA, TYR, PMEL*), fibroblasts (*PDGFRA, PDGFRB, FGF7, DCN, LUM*), monocytes/macrophages (*S100A12, CD14, CLEC10A, CD68, CD80, CD86, MRC1, CD163*), keratinocytes (*FLG, IVL, KRT1, KRT5, KRT10, KRT14*), endothelial cells (*VWF, PECAM1, SELE, CDH5*), smooth muscle cells (*CNN1, TAGLN, ACTA2, MYH11*), Schwann cells (*MPZ, PMP22, MAL, NGFR*), mast cells (*FCER1A, TPSB2, CMA1, TPSAB1*), sweat gland cells (*SCGB1D2, SCGB2A2, DCD, AQP5*), lymphatic endothelial cells (*LYVE1, PROX1, PDPN, FLT4*), and hair follicle cells (*KRT6B, SOX9, SFRP1, LHX2*).

### 2.6 Gene set enrichment analysis

Differential expression was performed in Seurat using the FindMarkers function. For each target cluster (e.g., Tex.CD4), we compared cells in the cluster against the specified reference population and identified genes detected in at least 25% of cells in either group (min.pct = 0.25). No log-fold-change prefiltering was applied (logfc.threshold = 0), such that all eligible genes were retained for downstream ranking.

For gene set enrichment, gene symbols were converted to Entrez Gene IDs using clusterProfiler::bitr with the human annotation database org.Hs.eg.db. The differential expression results were then joined with the ID mapping table, and genes were ranked by avg_log2FC in descending order. GO enrichment at the gene-set level was conducted using clusterProfiler::gseGO (Biological Process ontology, ont = “BP”). We used simple permutation-based enrichment with 10,000 permutations (nPermSimple = 10000) and set eps = 0 to avoid numerical issues. Gene sets were restricted to sizes between 5 and 500 genes (minGSSize = 5, maxGSSize = 500). Statistical significance was assessed with a nominal cutoff of p < 0.05 (pvalueCutoff = 0.05) and adjusted using the Benjamini–Hochberg method (pAdjustMethod = “BH”). In the Tex.CD4 subset, we focused in particular on immune-related pathways, including positive regulation of T cell activation and positive regulation of immune response.

### 2.7 Monocle2 analysis

To reconstruct differentiation trajectories within the CD4^+^ T-cell compartment, we subsetted the integrated Seurat object to include Naive.CD4, Th.CD4, Treg.CD4, and Tex.CD4 populations. The raw RNA count matrix and corresponding cell metadata were used to construct a Monocle2 CellDataSet (Monocle2 v2.3.0) with a negative binomial expression model (negbinomial.size()), and lowerDetectionLimit was set to 0.5. Genes were detected using detectGenes(min_expr = 0.1), and only genes expressed in at least 10 cells (num_cells_expressed ≥ 10) were retained for downstream trajectory inference. Size factors and dispersions were estimated using estimateSizeFactors() and estimateDispersions().

To define ordering genes for pseudotime reconstruction, we performed differentialGeneTest() with the model ~ cellType and selected genes with qval < 0.01 as candidates. For trajectory construction, we further prioritized the top 3,000 genes ranked by q-value and set them as ordering genes using setOrderingFilter(). Dimensionality reduction was conducted using DDRTree, followed by pseudotime ordering with orderCells().

To identify genes associated with lineage bifurcation between exhausted and Th-like CD4^+^ T-cell fates, we applied Branch Expression Analysis Modeling (BEAM) on the reconstructed trajectory. BEAM results were ranked by q-value, and highly branch-dependent genes were defined using a stringent threshold (qval < 1×10^−50^). These genes were visualized using plot_genes_branched_heatmap() with 6 gene modules (num_clusters = 6), with branch labels corresponding to Tex.CD4 and Th.CD4.

To interpret the biological functions of branch-associated gene programs, we performed GO-BP enrichment analysis for each BEAM-derived gene module identified from the branched heatmap annotation. For each module, gene symbols were converted to Entrez IDs using org.Hs.eg.db via clusterProfiler::bitr, and enrichment was conducted with clusterProfiler (v4.1.4) using Benjamini–Hochberg adjustment (pAdjustMethod = “BH”). Analyses were run with pvalueCutoff = 0.1, qvalueCutoff = 0.05, and minGSSize = 5. Key enriched biological functions from statistically significant GO terms were summarized and displayed alongside the corresponding gene modules in the branched heatmap.

### 2.8 Cell-cell communication analysis

Intercellular communication was evaluated using CellChat (v2.2.0) to infer putative signaling networks based on curated ligand–receptor interactions, with an emphasis on pathways relevant to T-cell immune regulation. Communication patterns across cell populations were summarized by comparing outgoing (sender) and incoming (receiver) signaling strengths and visualized using heatmaps. To specifically assess regulatory inputs to Tex.CD4, we performed a targeted analysis of signaling directed toward this subset to identify the major contributing cell populations and the dominant ligand–receptor pairs driving these interactions. Notably, monocytes/macrophages (MoMacs) were identified as a major signaling source influencing Tex.CD4. We therefore systematically summarized all ligand–receptor signaling pathways from MoMacs to Tex.CD4 to delineate the complete set of interactions mediating this effect.

### 2.9 Fungal strain

The fungal strain WH10-002, identified as *F. pedrosoi*, was obtained from a skin lesion of a CBM patient. Species identification was confirmed by sequencing the ITS1-5.8S rRNA-ITS2 region, and the sequence was deposited in GenBank under accession number GQ420654.1. To obtain hyphal material for study, the stock culture was transferred to Sabouraud dextrose broth (SDB; Difco™ Ref. 238220, BD, Sparks, MD) and incubated for two weeks at 28 °C.

### 2.10 *In vitro* induction of muriform cells

To induce the formation of muriform cells, the mycelia grown in SDB for 15 days were dispersed with a glass homogenizer, filtered through a nylon filter (200 mesh), and the resulting short hyphal fragments were adjusted to a final concentration of 0.5×106/mL. Then, 500 μL of the fragmented hyphae was then transferred into 30 mL of synthetic basal medium (ATCC medium 830, pH 5.5) with the following composition (g/L): NH_4_NO_3_, 1.5; MgSO_4_, 0.1; KH_2_PO_4_, 1.8; thiamine-HCl, 1.0×10^−4^; biotin, 5×10^−5^; and glycerol, 6.5. Nikkomycin Z (Sigma-Aldrich, N8028) was supplemented at a final concentration of 50 μg/mL. Cultures were incubated at 35 °C for 50 days, and muriform cell formation was observed by microscopic examination (23).

### 2.11 Murine model of subcutaneous dematiaceous fungal infection

Male C57BL/6n mice (6–8 weeks old, specific pathogen-free [SPF]) were obtained from the Animal Laboratory Center of Peking University First Hospital and maintained under SPF conditions. Mice were anesthetized by intraperitoneal injection of 0.4 μL Anasedan and 0.35 mL Dopalen per kg body weight before infection. Each mouse was injected with 100 μL of *F. pedrosoi* muriform cells at 1.5 × 10^8^ CFU/mL. Control mice received a subcutaneous injection of 100 μL normal saline (23).

### 2.12 Histopathology examination

Skin biopsy samples were obtained from the mouse footpads. All specimens were fixed in 10% formalin and then embedded in paraffin for histological processing. Sections of 3–5μm thickness were prepared, stained with hematoxylin and eosin (H&E), and evaluated under a microscope. Representative H&E-stained images at respective time points were then chosen from each group for further analysis.

### 2.13 Fluorescent multiplex immunohistochemistry, tissue imaging, and analysis

For multiplex immunohistochemistry (mIHC), formalin-fixed, paraffin-embedded (FFPE) tissue sections (2-5 μm thick) were prepared. Following deparaffinization in xylene (30 minutes) and a graded ethanol series for rehydration, the slides were washed and subjected to heat-induced epitope retrieval in boiling EDTA buffer (ZLI-9079, ZSBio) by use a microwave oven. After blocking with Antibody Diluent/Block (Alpha X Bio), staining was carried out on the AlphaXPainter X30 system (Alpha X Bio) utilizing four distinct antibody panels. The primary antibodies used were as follows: for CD4, human antibody ET1609–52 and mouse antibody Ab183685 were used; for TIM-3, human antibody CST45208S and mouse antibody Ab241332 were used; for PD-1, human antibody ZM0381 and mouse antibody Ab214421 were used; and for LAG-3, both human and mouse antibodies were 16616-1-AP.

All primary antibodies were incubated for 1 h at 37 °C, and then incubated with Alpha X Polymer HRP Ms+Rb (Alpha X Bio) at 37 °C. Alpha X 7-Color IHC Kit (AXT37100031, Alpha X Bio) was used for visualization. After each staining cycle, heat-induced epitope retrieval was performed to remove all the antibodies including primary & secondary bound antibodies. Slides were stained with DAPI for 5 minutes and mounted with Antifade Mounting Medium (I0052; NobleRyder). Images were scanned by ZEISS AXIOSCAN 7 and analyzed by HALO software (v3.6).

### 2.14 Statistical analysis

Data were analyzed using GraphPad Prism software, and the results are expressed as mean ± standard error (SE). The Mann–Whitney U test was applied to assess statistical significance between groups. A p-value < 0.05 was considered statistically significant.

## 3 Results

### 3.1 Single-cell transcriptomics defines the immune cellular landscape of chromoblastomycosis lesions

**Figure 1:**
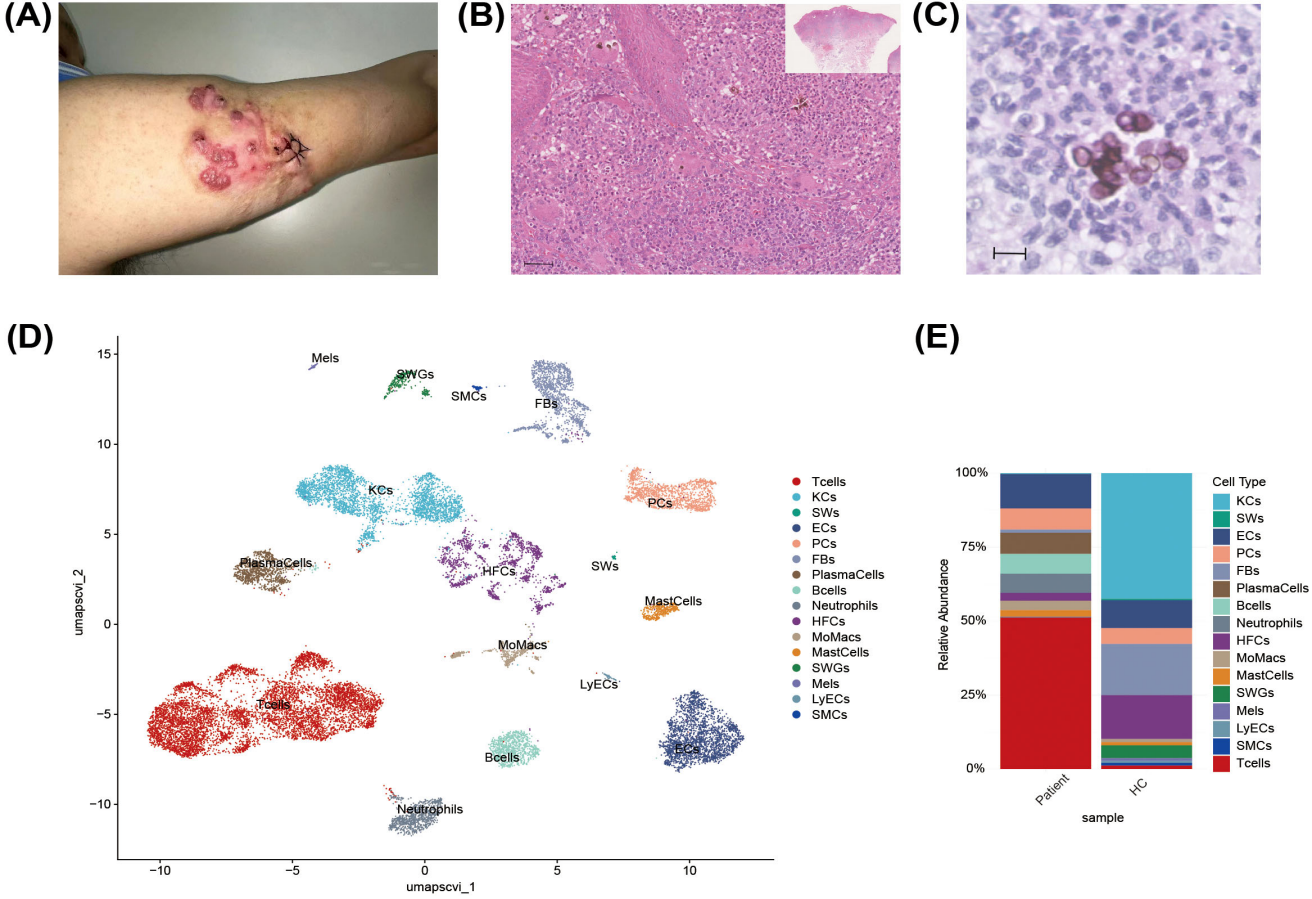
Clinical features and cell type annotation of skin lesion in a patient with CBM: **(A)** photograph of a skin lesion on the arm of patient with chromoblastomycosis, showing typical verrucous erythema and nodules. **(B)** Hematoxylin and eosin staining of the lesional tissue, demonstrating a chronic inflammatory granulomatous response. (20× objective lens, Scale bar: 50 μm). **(C)** A higher-magnification view of Periodic Acid-Schiff staining of the lesional tissue, showing thick-walled sclerotic cells (muriform bodies). (40× objective lens, Scale bar: 10 μm). **(D)** Uniform Manifold Approximation and Projection (UMAP) plot showing the annotated cell types. KCs, keratinocyte; SWs, Schwann cells; ECs, endothelial cells; PCs, pericytes; FBs, fibroblasts; HFCs, hair follicle cells; MoMacs, monocytes/macrophages; SWGs, sweat gland cells; Mels, melanocytes; LyECs, lymphatic endothelial cells; SMCs, smooth muscle cells. **(E)** Stacked bar plot displaying the compositional differences of cell types between patient and healthy control (HC) samples.

The patient was a 43-year-old male who had developed multiple red nodules on his left upper limb nine years earlier. The lesions appeared as dark-red nodules with surface crusting and exudation (**Figure 1A**). Fungal culture identified *C. carrionii* as the causative pathogen. Histopathological examination with HE staining revealed pseudoepitheliomatous hyperplasia of the epidermis and diffuse mixed inflammatory cell infiltration throughout the dermis, predominantly composed of lymphocytes, plasma cells, multinucleated histiocytes, and neutrophilic microabscesses (**Figure 1B**). High-power microscopy revealed the pathognomonic sclerotic bodies (**Figure 1C**). The patient was diagnosed with CBM and had been maintained on long-term oral itraconazole (200 mg/day) and terbinafine (250 mg/day), in addition to undergoing surgical excision of local lesions. However, his skin lesions persisted and remained refractory to treatments. To explore the reason of chronicity of CBM, we performed scRNA-seq on lesional tissue from this patient. The analysis showed a highly diverse immune infiltrate in the lesion (**Figure 1D**). In the skin lesions of patient, T cells constituted the dominant infiltrating population, accompanied by substantial proportions of macrophages/monocytes, plasma cells, and neutrophils (**Figure 1E**). However, there was very low percentage of T cells in the healthy control. This composition reflects an inflammatory microenvironment driven by both adaptive and innate immune responses.

### 3.2 Exhausted CD4+ T cells emerge as the predominant T cell subset in chronic chromoblastomycosis lesions

**Figure 2:**
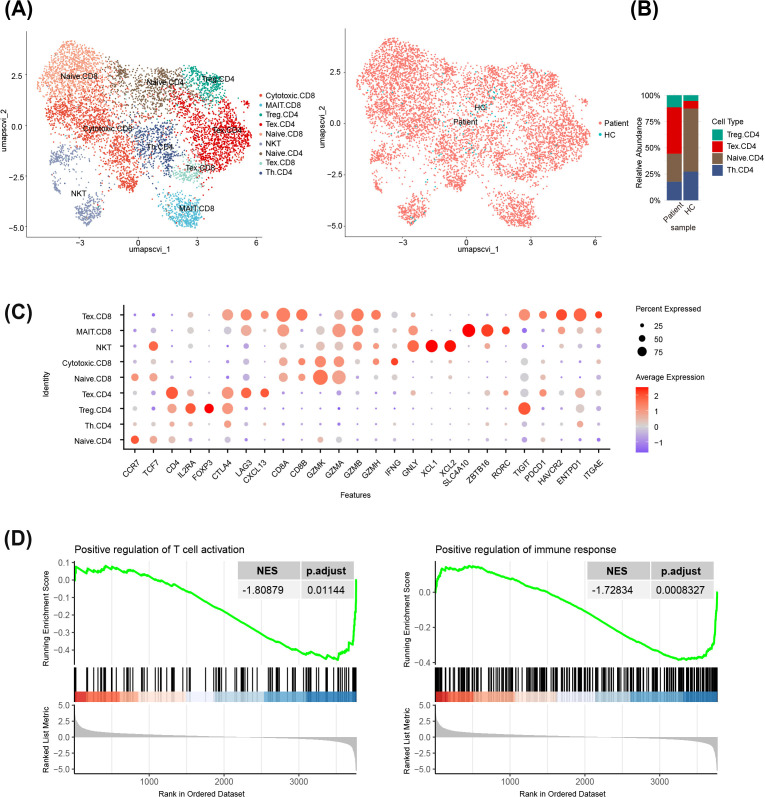
Single-cell analysis of CD4+ T cell subsets in CBM: **(A)** UMAP visualization illustrating the annotated T cell subsets (left) and the distribution of cells from patient and HC samples (right).Treg, regulatory T cell. Th, T helper cell. **(B)** Stacked bar plot showing the proportional differences of CD4^+^ T cell subsets between patient and HC samples. **(C)** Bubble plot displaying the expression of specific molecular markers associated with distinct T cell states. **(D)** GSEA revealed a significant enrichment of pathways for the positive regulation of T cell activation (left) and immune response (right) in exhausted T cells.

To further investigate the characteristics of the immune microenvironment of CBM, we conducted an in-depth analysis of T lymphocytes using scRNA-seq data from the patient’s lesional skin. Based on signature gene expression, T cells were classified into four distinct CD4^+^ T cell subsets, four CD8^+^ T cell subsets, and NKT cells, revealing highly transcriptional heterogeneity. The overall number of T cells was significantly increased in the patient’s lesion compared to the healthy control (**Figure 2A**). Quantitative analysis demonstrated a marked increase in the proportion of exhausted CD4^+^ T cells and a mild elevation in regulatory T cells. In contrast, other subsets, such as naïve T cells and T helper cells, were relatively reduced (**Figure 2B**). These findings indicated that exhausted CD4^+^ T cells constituted the most prominent infiltrating T cell subset and exhibited high expression of significant exhaustion markers such as CTLA4 and LAG3 (**Figure 2C**). Further analysis of the exhausted CD4^+^ T cell population revealed a low expression of canonical lineage-specifying markers (such as TBX21, IFNG, GATA3, IL4, RORC, and IL17A), consistent with the fundamental biology of T cell exhaustion (**Supplementary Figure 1**). GSEA of the top 50 genes upregulated in exhausted CD4^+^ T cells revealed significant suppression of immune activation pathways, including “Positive regulation of T cell activation” (NES = -1.809, p.adjust = 0.011) and “Positive regulation of immune response” (NES = -1.728, p.adjust = 0.001) (**Figure 2D**). These data support the presence of severe CD4^+^ T cell exhaustion within chronic CBM lesions.

### 3.3 Pseudotime trajectory analysis reveals a developmental pathway towards T cell exhaustion

**Figure 3:**
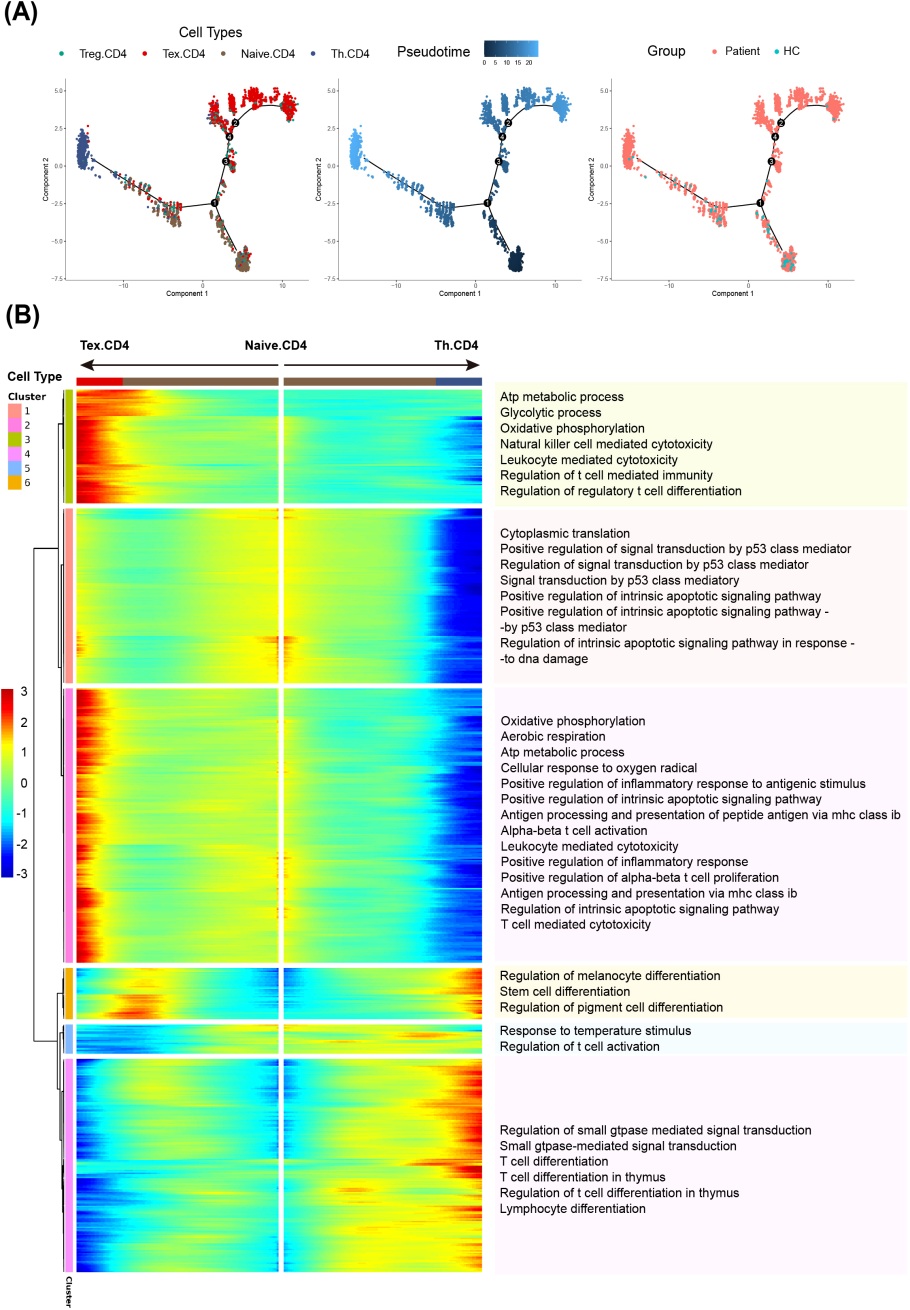
Developmental trajectory and functional regulation of CD4^+^ T cell subsets in CBM: **(A)** Monocle2 analysis reveals the developmental trajectory of CD4^+^ T cell subsets (left), the predicted starting point and direction of differentiation (middle), and the distribution of cells from patient and HC samples along the pseudotime trajectory (right). **(B)** BEAM analysis identifies key branch-dependent genes. The heatmap displays gene expression patterns as naïve T cells differentiate towards exhausted or Th-like CD4^+^ T cell fates. Significantly enriched pathways for genes upregulated (red) or downregulated (blue) along the exhausted CD4^+^ T cell branch are listed, with gene counts for each pathway indicated.

To investigate the differentiation pathways and functional evolution of CD4^+^ T cells in CBM, we performed pseudotemporal analysis of CD4^+^ T cells. The reconstructed trajectory originated from naïve CD4^+^ T cells, branching toward helper T cells and exhausted T cells (**Figure 3A**). Compared with healthy controls, patient-derived T cells were markedly enriched in the terminal exhausted region of the trajectory. This discover suggests that the chronic infectious microenvironment drives progressive differentiation toward T cell exhaustion.

Based on distinct gene expression patterns, we identified CD4^+^ T cells into six clusters, each with unique cellular pathway activation profiles (**Figure 3B**). Genes in Clusters 2 and 3 were highly expressed in Tex. CD4 cells but showed lower expression in functional Th. CD4 cells. These clusters are enriched with pathways associated with ATP metabolism, glycolysis, and oxidative phosphorylation. Cluster 1 genes were also specifically upregulated in Tex.CD4 cells modestly and were associated with p53-mediated signal transduction and activation of intrinsic apoptotic signaling pathways, suggesting that exhausted T cells undergo substantial cellular stress and activation of apoptotic programs. In contrast, genes in cluster 4 and 5 were most highly expressed in Th.CD4 cells, with lower expression levels observed in exhausted CD4^+^ T cells. These clusters included genes central to immune effector functions, such as regulation of T cell activation and leukocyte-mediated cytotoxicity which is consistent with the core functional role of Th cells. The pseudotime trajectory analysis demonstrates that Tex.CD4 cells in CBM lesions exhibit a dysfunction profile, with downregulation of genes involved in immune effector activity (Clusters 4 and 5) and aberrant activation of pathways associated with energy metabolism pathways (Clusters 2 and 3). These findings indicate that chronic infection in CBM drives CD4^+^ T cells toward functional exhaustion, accompanied by significant metabolic reprogramming and immune dysfunction.

### 3.4 MoMacs are likely a major contributor to CD4+ T cell exhaustion via inhibitory receptor engagement

**Figure 4:**
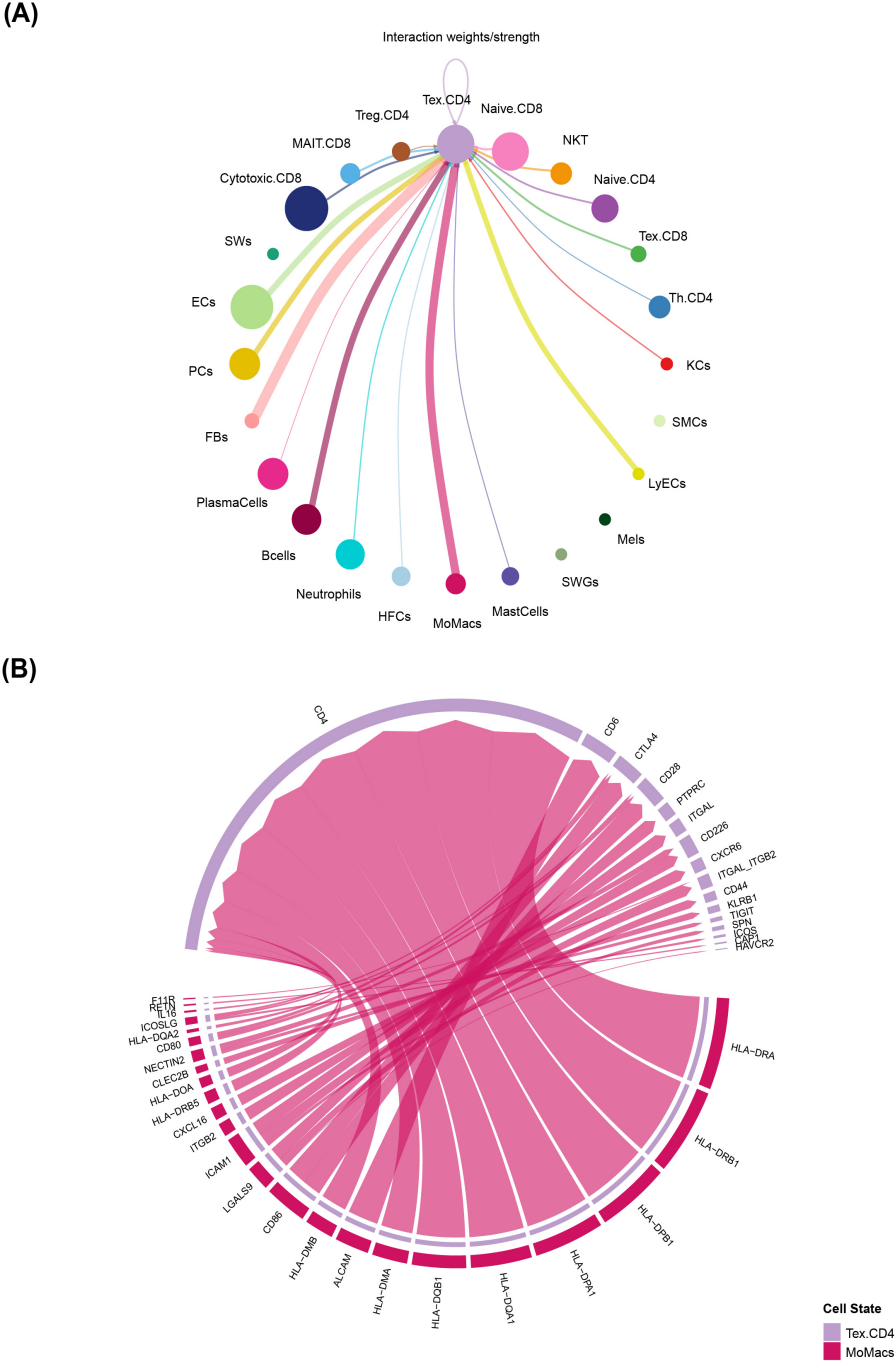
Cell communication analysis of exhausted CD4^+^ T cells: **(A)** Circle plot details the cell subsets sending signals to exhausted CD4^+^ T cells while the thickness of the line represents interaction strength. **(B)** Circle plot details the main ligand-receptor pathways MoMacs sending signals to exhausted CD4^+^T cells.

To explore the complex cell–cell communication network within the CBM lesion microenvironment, we applied the CellChat algorithm to analyze ligand–receptor interactions among major cell populations. We focused on the interaction strength between exhausted CD4^+^ T cells and other cell populations within the CBM lesion microenvironment. We discovered that Tex.CD4 cells exhibited strong interactions with several cell types, including monocytes/macrophages (MoMacs), fibroblasts (FBs) and keratinocytes (KCs). The most prominent interaction weight was observed between Tex.CD4 cells and MoMacs (**Figure 4A**), indicating that MoMacs may play a significant role in driving the chronic exhaustion of CD4^+^ T cells.

To further investigate this, we analyzed the ligand-receptor pairs mediating the communication between MoMacs and Tex.CD4 cells, several key pairs were identified with high interaction strength (**Figure 4B**). The most dominant interactions involved HLA with CD4, consistent with persistent antigen presentation. Other significant pairs included ALCAM-CD6, CD86-CTLA4, LGALS9-CD44/CD45, LGALS9-HAVCR2 and NECTIN2-TIGIT. These receptor-ligand pairs, particularly the inhibitory pathways, have been previously established in the literature to contribute to T cell dysfunction and exhaustion (24–28). This analysis reveals that MoMacs are the dominant communicating partners of exhausted CD4^+^ T cells in CBM lesions, primarily via sustained antigen presentation and multiple co-inhibitory signals, which may provide a mechanistic basis for the dysfunctional T cell state that characterizes this chronic infection.

### 3.5 Multiplex immunofluorescence validates the accumulation of exhausted CD4^+^ T cells in human CBM

**Figure 5:**
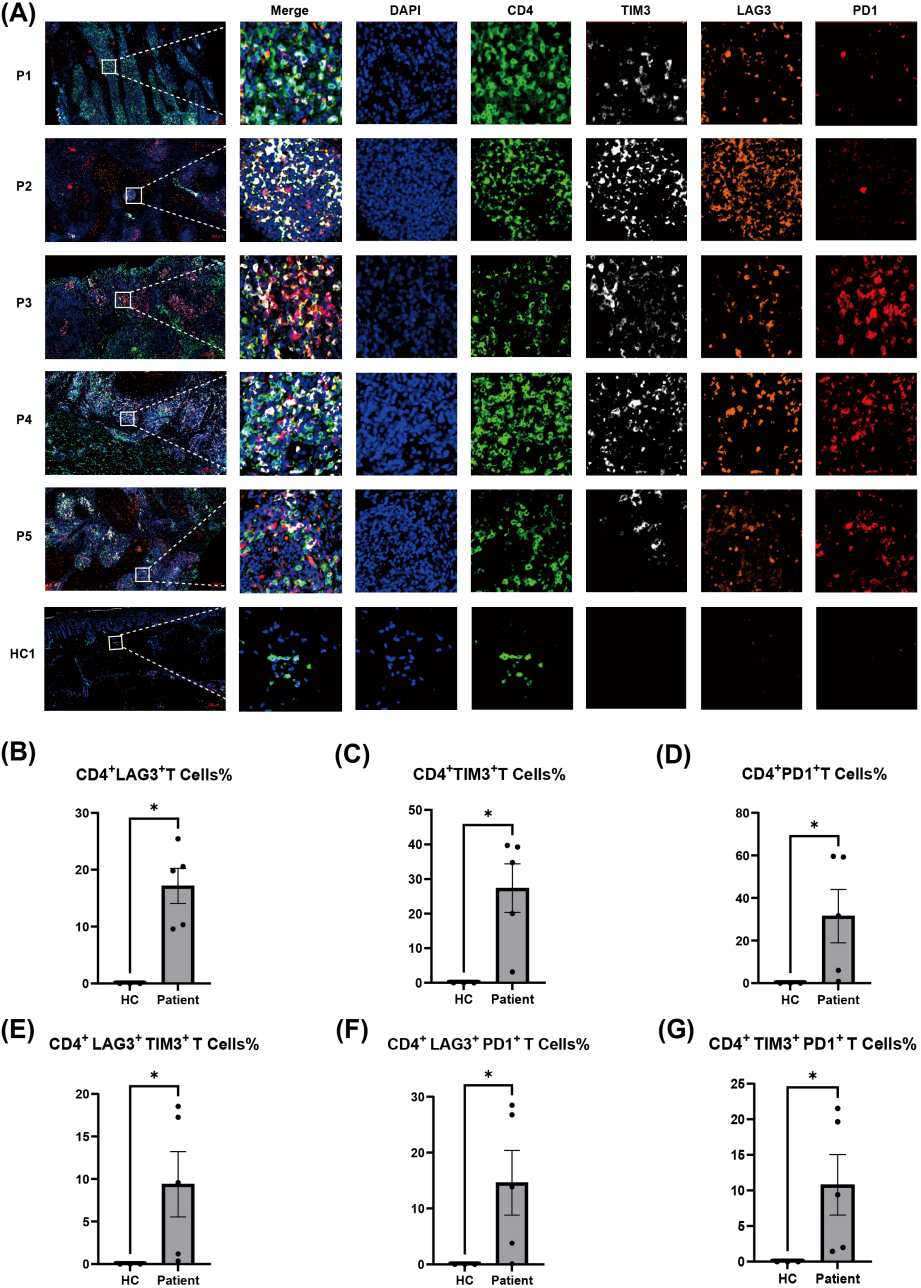
Co-expression of immune checkpoint molecules on CD4^+^ T cells in lesions of chromoblastomycosis patients: **(A)** Representative multiplex immunofluorescence images showing the expression and co-localization of CD4 (green), TIM3 (white), LAG3 (orange), and PD1 (red) in lesional skin from five chromoblastomycosis patients (P1-P5) and normal skin from one healthy control (HC1). Nuclei were counterstained with DAPI (blue). (20× objective lens, Scale bar: 200 μm). **(B–G)** Quantitative analysis of CD4^+^ T cell subsets co-expressing immune checkpoint molecules within the lesions: **(B)** Percentage of CD4^+^LAG3^+^ T cells among all the CD4^+^ T cells. **(C)** Percentage of CD4^+^TIM3^+^ T cells among all the CD4^+^ T cells. **(D)** Percentage of CD4^+^PD1^+^ T cells among all the CD4^+^ T cells. **(E)** Percentage of CD4^+^LAG3^+^TIM3^+^ T cells among all the CD4^+^ T cells. **(F)** Percentage of CD4^+^LAG3^+^PD1^+^ T cells among all the CD4^+^ T cells. **(G)** Percentage of CD4^+^TIM3^+^PD1^+^ T cells among all the CD4^+^ T cells. Data are presented as mean ± SE. * p < 0.05, by Mann–Whitney U test. HC, healthy control.

To validate the findings of scRNAseq, we used multiplex immunohistochemistry to assess the accumulation of exhausted CD4^+^ T cells in skin lesion samples from five patients with CBM (**Figure 5A**). These patients were infected by either *F. monophora* or *C. carrionii*, detailed clinical profiles are provided in **Supplementary Table 1**. We found marked co-localization of the immune checkpoint molecules, TIM3, LAG3, and PD1 with CD4 in CBM lesions. Compared with healthy controls, CBM lesions exhibited significantly higher densities of TIM-3–, LAG-3–, and PD-1–positive cells.

We observed substantial populations of CD4^+^ T cells expressing individual checkpoint molecules, as well as subsets co-expressing two or even three markers, indicating a deeply exhausted phenotype (**Figures 5B–G**). The increase in LAG-3^+^ cells was the most pronounced and consistent difference between CBM lesions and healthy controls. In contrast, while PD-1 expression was significantly elevated overall in the patient group, it showed considerable variation in its density, indicating heterogeneous PD-1 expression across individuals. This result strongly corroborates the transcriptomic identification of a dominant exhausted CD4^+^ T cell subset in the CBM microenvironment.

### 3.6 A murine model of chromoblastomycosis recapitulates progressive T cell exhaustion with disease chronicity

**Figure 6:**
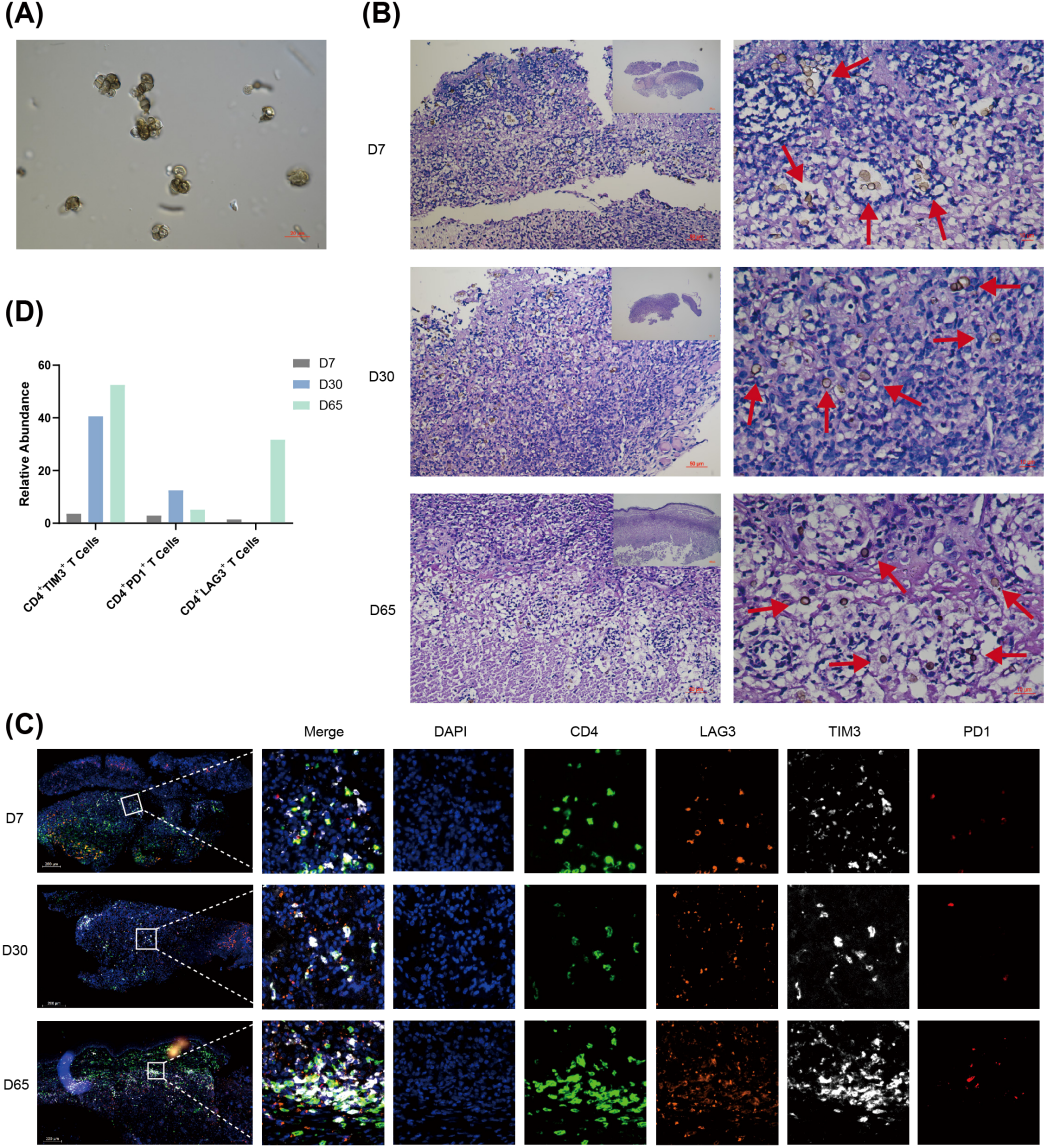
Dynamic evolution of CD4^+^ T cell exhaustion in a murine model of chromoblastomycosis: **(A)** *In vitro*-induced muriform cells of *Fonsecaea pedrosoi*. (60× objective lens, Scale bar: 20 μm). **(B)** H&E staining of footpad sections from mice infected with *Fonsecaea pedrosoi* at days 7, 30, and 65 post-infection, showing inflammatory responses and histopathological changes. (20× and 40× objective lens, Scale bar: 50 and 10 μm). **(C)** Representative multiplex immunofluorescence images of lesional tissues at different time points. Staining shows the co-localization of CD4 (green), LAG3 (orange), TIM3 (white), and PD1 (red). Nuclei were counterstained with DAPI (blue). (20× objective lens, Scale bar: 200 μm). **(D)** Quantitative analysis of the relative abundance of CD4^+^LAG3^+^, CD4^+^TIM3^+^, CD4^+^PD1^+^ cells among all the CD4^+^ T cells from the images in **(C)** (n =1).

To investigate host immune responses to CBM, we induced sclerotic cells *in vitro* (**Figure 6A**) and inoculated them into the footpads of C57BL/6n mice to establish a murine infection model. Lesional tissues were collected at 7, 30, and 65 days post-infection for HE staining and multiplex immunofluorescence analysis. HE staining confirmed the presence of characteristic muriform cells (**Figure 6B**). Multiplex immunofluorescence analysis revealed significant co-localization of the immune checkpoint molecules TIM-3, LAG-3, and PD-1 with CD4-positive cells in the skin lesions (**Figure 6C**). At different time points during the chronic infection, the expression patterns of these immune checkpoints varied. The proportion of CD4^+^TIM3^+^ T cells showed a progressive increase, rising from 3.63% at day 7 to 40.65% at day 30, and further to 52.52% by day 65. In contrast, CD4^+^PD1^+^ T cells displayed a different pattern. While their frequency increased from 2.94% at day 7 to 12.50% at day 30, it subsequently declined to 5.17% at day 65. This late-stage decrease aligns with observations of heterogeneous PD-1 expression in human lesions. The proportion of CD4^+^LAG3^+^ T cells remained similar between days 7 and 30, but was elevated to 31.70% at day 65 (**Figure 6D**). These findings indicate a more pronounced immunosuppressive microenvironment in the late phase of chronic infection.

## 4 Discussion

CBM, a neglected tropical disease, is primarily caused by dematiaceous fungi, such as *F. pedrosoi, F. monophora* and *C. carrionii*. The disease follows a chronic and refractory course, often progressing to extensive scarring, ulceration, and joint deformities in advanced stages. However, the mechanism underlying the chronicity of CBM remains unclear. This study provides the systematic characterization of the immune landscape in CBM lesions at single-cell resolution and identifies CD4^+^ T-cell exhaustion as a significant mechanism underlying disease chronicity. This finding offers a refined understanding of CBM immunopathology, and provides a theoretical basis for the development of targeted immunotherapeutic strategies.

The lesional tissue analyzed in this study was from a patient with a nine-year history of refractory CBM, representing a classic and severe case of this chronic infection. To explore the cellular basis of CBM, we performed single-cell RNA sequencing (scRNA-seq) on the patient’s lesional skin. Our analysis revealed a profoundly altered immune landscape compared to healthy control skin. We observed a marked expansion of inflammatory cells, with T lymphocytes constituting the dominant population, accompanied by substantial infiltrates of macrophages/monocytes, plasma cells, and neutrophils. The increased inflammatory populations directly reflect the sustained immune activity and is consistent with the histopathological features of persistent and refractory infection.

The most significant finding of this study is the identification of a distinct exhausted CD4^+^ T cell subset within CBM lesions. This population exhibits classic exhaustion characteristics: high expression of multiple inhibitory receptors (e.g., PD-1, TIM-3, LAG-3) and downregulation of effector functions. Similar dysfunctional states have been described in T cells during chronic infections such as HIV, HCV, and HBV (29–31). Previous studies in fungal infections have also reported an increase in exhaustion markers in invasive aspergillosis (32, 33) and CBM (34). However, most of these investigations were limited primarily to PD-1 and did not specifically focus on the distinct exhausted CD4^+^ T cell subset. Our study provides the confirmation of CD4^+^ T cell exhaustion in CBM and expands the recognized scope of this phenomenon. Unlike viral infections and cancer, T cell exhaustion caused by fungal infection may involve unique regulatory mechanisms and characteristics, which provides a new direction for further research.

The development of T cell exhaustion in CBM involves multiple functional changes. Our study reveals significant metabolic reprogramming in exhausted T cells in CBM. It has been reported that altered expression of genes encoding key metabolic pathways, including glycolysis and oxidative phosphorylation, may lead to cellular energy metabolism disorders, ultimately impairing effector functions (35, 36). A study in HCV infection have reported that virus-specific exhausted CD8^+^ T cells display a marked upregulation of transcription associated with impaired glycolytic and mitochondrial functions, which are linked to enhanced p53 signaling (37). This exhausted state severely impacts anti-fungal immunity. Exhausted T cells exhibit reduced capacity to produce effector cytokines and clear pathogens. Conversely, exhausted T cells may further suppress the function of surrounding immune cells through expression of inhibitory molecules.

Our interaction analysis reveals that monocytes/macrophages (MoMacs) primarily function as signal senders, while exhausted CD4^+^ T cells functioned as the receivers. We found that MoMacs interact with CD4^+^ T cells through persistent antigen presentation with multiple ligand pairs, including CD86-CTLA4, LGALS9-CD44/CD45, LGALS9-HAVCR2, and NECTIN2-TIGIT. The co-inhibitory pathways, such as CTLA4-CD86 and NECTIN2-TIGIT, have been extensively proved to correlate with T cell exhaustion. The acquired upregulation of inhibitory immune checkpoints can induce T cell exhaustion, which often limits the efficacy of monotherapy with immune checkpoint inhibitors (24). Galectin-9 has recently been described as a key driver of T cell dysfunction. In chronic lymphocytic leukemia, the galectin-9–TIM-3 axis was identified as a central mediator of T cell exhaustion and a potential immunotherapeutic target (24, 26, 27, 38). Our data suggests that significant LGALS9-related interactions between MoMacs and exhausted CD4^+^ T cells in CBM. MoMacs may similarly induce CD4^+^ T cell exhaustion via this pathway, which corresponds the mechanism observed in malignant environments (25). We also observed active ALCAM–CD6 interaction between MoMacs and exhausted CD4^+^ T cells. Although ALCAM-CD6 typically provides a co-stimulatory adhesion signal, persistent stimulation in a chronic disease can also lead to T cell dysfunction. This aligns with emerging evidence from tumors (28). In bladder cancer, the CD6/ALCAM-mediated pathway is associated with an immunosuppressive microenvironment and disease recurrence (39). Our findings delineate MoMacs linked to CD4^+^ T cell exhaustion through persistent antigen presentation coupled with several inhibitory signals and dysregulated co-stimulation.

Our multiplex immunofluorescence analysis confirmed a significant accumulation of exhausted CD4^+^ T cells within CBM lesions, as evidenced by markedly elevated co-localization of the canonical exhaustion markers LAG-3, PD-1, and TIM-3 with CD4^+^ T cells compared to healthy control skin. Among these markers, the upregulation of LAG-3 was the most pronounced and consistent across patient samples, establishing itself as a central and reliable biomarker of T cell exhaustion in the CBM microenvironment. In contrast, PD-1 expression exhibited considerable individual variability. This heterogeneity in PD-1 levels may due to multiple factors, such as disease course variations and prior treatment history. PD-1 is a dynamic checkpoint often associated with recent activation and its expression may be more susceptible to variable antigen load or transient inflammatory signals (40). The distinct expression patterns of different immune checkpoints suggest that, despite the activation of multiple inhibitory pathways, their relative contributions may vary. This underscores the complexity of the exhausted T cell phenotype in CBM.

To validate our findings, we utilized a murine model to observe the development of exhausted T cells and disease progression in CBM. The murine model induced by *F.pedrosoi* was selected for its status as the most established and reproducible system, closely mimicking human pathology through the induction of characteristic muriform cells and chronic granulomatous lesions (23). It is important to note that, the current data are derived from a limited sample per time point, which restricts statistical analysis and the generalizability of the quantitative expression levels.​ By monitoring immune checkpoints at different time points, we discovered that T cell exhaustion is a progressive process. In the early stage, TIM-3 expression was markedly elevated. This was followed by a further upregulation of both TIM-3 and LAG-3 in the late stage. However, PD-1 expression displayed a lower overall magnitude of change compared to TIM-3 and LAG-3. This relative variability in PD-1 levels aligns with the inter-individual heterogeneity observed in human CBM lesions. The distinct temporal expression profiles of these checkpoint molecules suggest that they may perform different functions. TIM-3 may play a key role in initiating exhaustion, while the elevation of TIM-3 and LAG-3 appears to be crucial in maintaining the terminal exhausted phenotype. This dynamic expression profile provides new insights for the optimal timing of clinical intervention.

Previous studies have indicated the important role of CD4^+^ T cell differentiation in CBM (41, 42). And it has also been reported that the expression of PD-1 and PD-L1 is upregulated in chronic CBM, yet the specific cell types expressing these inhibitory molecules remain unconfirmed (34). We applied single-cell technology to identify a distinct population of exhausted T cells in CBM tissue and systematically analyzed the expression profiles of multiple exhaustion-related markers (PD-1, LAG-3, TIM-3) for the first time. Functional analysis revealed that this cell population does not express classic Th cell-associated transcription factors or effector molecules, which aligns with its loss of effector function and corresponds to the chronic progression of CBM. Furthermore, we elucidated the functional properties of these exhausted T cells, their interactions with other immune cells, and their pivotal role in disease chronicity of CBM.

This study systematically characterizes T-cell exhaustion in CBM by integrating single-cell transcriptomics, patient validation, and *in vivo* models. Importantly, our murine model successfully recapitulates the developmental trajectory of T-cell exhaustion. These findings not only deepen the understanding of CBM immunopathology but also provide insights for research on other chronic infectious diseases. Based on these insights, future studies may explore targeting T-cell exhaustion-related signaling pathways as an adjuvant therapeutic strategy for CBM.

## Data Availability

The datasets analyzed for this study can be found on the GSA for Human platform, Accession number: HRA017901.
