# Supplementary material for: Single-cell RNA sequencing unravels T cell exhaustion underlying the chronicity of chromoblastomycosis
Source: Front Immunol. 2026 Apr 17;17:1784450. doi: 10.3389/fimmu.2026.1784450 (PMC13133555; doi:10.3389/fimmu.2026.1784450)
Supplement: Supplementary file 1 [file DataSheet1.docx]

**
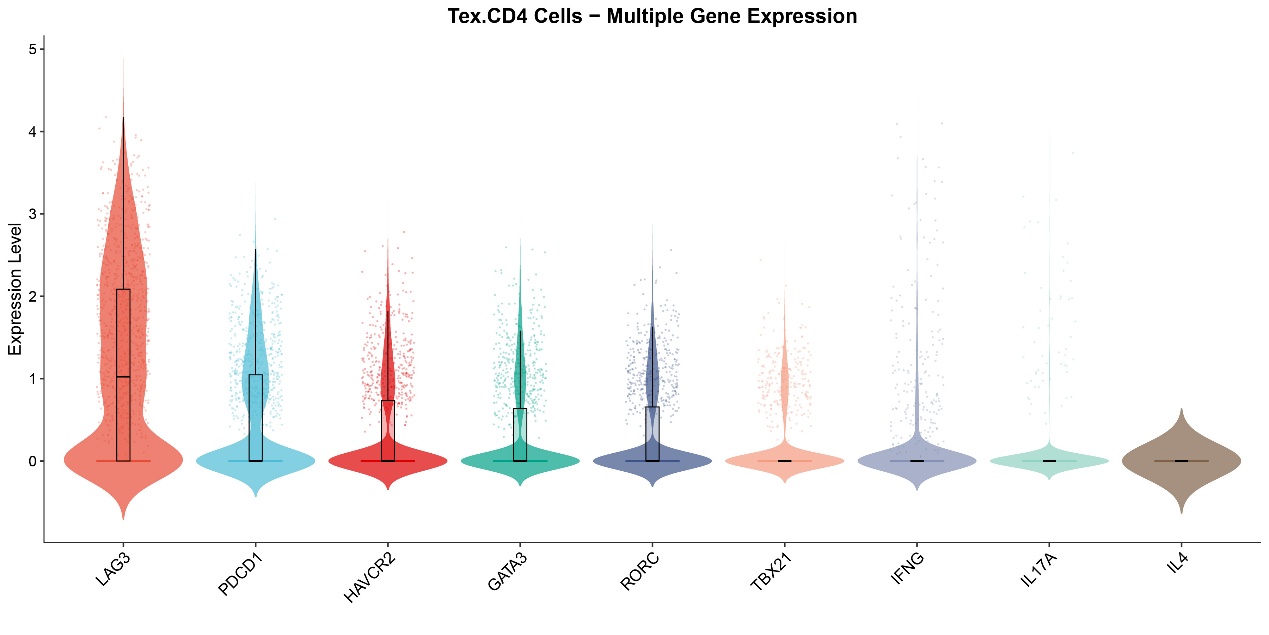
**

**Supplementary Figure S1. Expression patterns of immune-related genes in Tex.CD4 cells.**

Violin plots showing the expression distribution of nine selected genes in Tex.CD4 cells. Each violin represents the probability density of gene expression, with the width indicating the frequency of cells at a given expression level. Boxplots inside the violins show the median (center line), interquartile range (box bounds), and whiskers extending to 1.5× the interquartile range. Jittered points represent individual cells, with transparency (alpha = 0.3) to visualize density. Genes are ordered on the x-axis from left to right by decreasing mean expression level. Tex.CD4, exhausted CD4+ T cells.

**Supplementary table 1.** **Clinical characteristics of patients with CBM**

| Clinical characteristics of patients with CBM | | | | | | | | |
| --- | --- | --- | --- | --- | --- | --- | --- | --- |
| ID | Age(y) | Sex | Duration of infection(y) | Pathogenic fungus | Biopsy location | Therapy received at the time of biopsy | Co-existing illnesses | CARD9 mutations |
| P1 | 77 | M | 3 | *Cladophialophora carrionii* | Left arm | Itraconazole po. (200mg/day) | No | No |
| P2 | 46 | M | 12 | *Cladophialophora carrionii* | Left arm | Terbinafine po. (250mg/day) Itraconazole po. (200mg/day) | No | No |
| P3 | 63 | F | 3 | *Cladophialophora carrionii* | Left lower limb | Itraconazole po. (400mg/day) | No | No |
| P4 | 49 | F | 18 | *Fonsecaea monophora* | Left foot | Itraconazole po. (400mg/day) | No | No |
| P5 | 52 | M | 20 | *Fonsecaea monophora* | Left hand | Itraconazole po. (400mg/day) | No | No |

**Supplementary Methods**

**Gene expression analysis**

Single-cell RNA sequencing data were analyzed using the Seurat package (version 4.0) in R (version 4.1.0). To examine the expression patterns of immune-related genes in Tex.CD4 cells, we subsetted the Tex.CD4 cell population using the WhichCells function. The expression levels of nine selected genes (*PDCD1, LAG3, HAVCR2, TBX21, IFNG, GATA3, IL4, RORC, and IL17A*) were extracted using the FetchData function. Data were transformed into long format using the pivot_longer function from the tidyr package. Violin plots were generated using ggplot2 (version 3.3.5) to visualize the distribution of gene expression, with overlaid jittered points representing individual cells. Genes were ordered on the x-axis based on their mean expression levels (descending order).
